# Trends in cardiac rehabilitation enrollment post-coronary artery bypass grafting upon implementation of automatic referral in Southeast Asia: A retrospective cohort study

**DOI:** 10.34172/jcvtr.2022.22

**Published:** 2022-06-28

**Authors:** Karen V. Miralles-Resurreccion, Sherry L. Grace, Lucky R. Cuenza

**Affiliations:** ^1^Section of Cardiac Rehabilitation, Philippine Heart Center, Quezon City, Philippines; ^2^York University, Toronto, Ontario, Canada; ^3^KITE-Toronto Rehabilitation Institute & Peter Munk Cardiac Centre, University Health Network, University of Toronto, Ontario, Canada

**Keywords:** Automatic Referral, Cardiac Rehabilitation, Enrollment, Health Services Utilization, Low and Middle-Income Country

## Abstract

***Introduction:*** Cardiac rehabilitation (CR) is an effective but underutilized intervention. Strategies have been identified to increase its use, but there is paucity of data testing them in low-resource settings. We sought to determine the effect of automatic referral post-coronary artery bypass graft (CABG) surgery on CR enrollment.

***Methods:*** This is a retrospective cohort study assessing cardiac patients referred to CR at a tertiary center in Southeast Asia from 2013 to 2019. The paper-based pathway was introduced at the end of 2012. The checklist with automatic CR referral on the third day post-operation prompted a nurse to educate the patient about CR, initiate phase 1 and encourage enrollment in phase 2. Patients who were not eligible for the pathway for administrative or clinical reasons were referred at the discretion of the attending physician (i.e., usual care). Enrollment was defined as attendance at≥1 CR visit.

***Results:*** Of 4792 patients referred during the study period, 394 enrolled in CR. Significantly more patients referred automatically enrolled compared to usual care (225 [11.8%] vs. 169 [5.8%]; OR=2.2, 95% CI=1.8-2.7), with increases up to 23.4% enrollment in 2014 (vs. average enrollment rate of 5.9% under usual referral). Patients who enrolled following automatic referral were significantly younger and more often employed (both *P*<0.001); no other differences were observed.

***Conclusion:*** In a lower-resource, Southeast Asian setting, automatic CR referral is associated with over two times greater enrollment in phase 2 CR, although efforts to maintain this effect are required.

## Introduction

 Cardiac rehabilitation (CR) is a comprehensive secondary prevention intervention.^1^ CR participation reduces all-cause mortality by 13-26% and cardiac rehospitalizations by 31%.^2,3^Additionally, it is safe^4^ and cost-effective.^5^ Accordingly, the American Heart Association and American College of Cardiology Foundation gave CR the highest level of recommendation (Class I level of evidence A) for patients who have undergone coronary artery bypass grafting (CABG),^6^among other indications.

 Despite these benefits, CR utilization remains low.^4,7-11^ Physician referral is required for patients to access these programs, and then patients must attend to enroll. Based on “Get With the Guidelines” data from the United States (US), referral rates at 156 hospitals were 74% after CABG.^12^ With regard to enrollment, the US reports 24% (up from 16-19% in 1997)^8^ in 2011^13^ and 27% in 2017^14^ in indicated Medicare beneficiaries ≥ 65 years. Meta-analyses of published literature reporting CR utilization rates found overall referral rates at 43%^15^ and enrollment at 42%.^16^ Unfortunately in low-resource settings where the burden of cardiovascular disease is worst, CR utilization is even poorer.^17-19^ A three-year retrospective study in a tertiary hospital in the Philippines showed that among eligible patients, only 16% enrolled in phase 2 CR.^9^

 Multi-level barriers to CR referral and enrollment have been identified.^4,18-23^ Globally, lack of patient referral is the second greatest barrier to CR access, after lack of financial resources.^18^ There are now proven interventions to increase CR referral and enrollment.^7,24^ Chiefly, automatic or systematic referral, whereby clinical pathways or electronic health records are used to trigger CR referral^25^ results in significantly greater referral and enrollment rates when compared with usual referral.^7,10,11,26-29^ Enrollment is also greatly augmented through liaison discussions wherein clinicians discuss CR with patients at the bedside and encourage them to attend.^30,31^

 However, most of the studies in this area have been undertaken in high-income countries, where CR barriers are lower.^19^ Thus, there is paucity of data on the implementation and effects of automatic referral in low-resource, non-western settings.^32^ This study aimed to investigate the effect of instituting clinical pathway-based automatic referral with bedside CR encouragement post-CABG surgery on CR enrollment over time, in comparison to usual referral in the Philippines. Second, whether inequities in sociodemographic and clinical characteristics of enrollees were mitigated by the CR utilization intervention was explored.

## Materials and Methods

###  Setting

 The Philippines, located in Southeast Asia, is a middle-income country according to the World Bank. The Philippine Heart Center (PHC) is a large, tertiary care hospital in Manila. Its CR Section has been in the forefront of CR practice since 1975.

 The automatic referral process in this institution makes use of the Z Benefit Package,^33^ a program launched by Philippine Health Insurance Corporation (Philhealth) in 2012, to deal with illnesses considered debilitating or those considered to be financially and medically catastrophic, including CABG. Approval for patients to avail of the benefit is based on the following selection criteria: age 19-70 years, stable coronary artery disease requiring elective isolated CABG, not in severe decompensated heart failure (NYHA IV), not with severe angina (CCS Class III), no other cardiovascular procedures, no previous cardiac surgery, no previous angioplasty or stenting, EUROSCORE II and/or STS scoring predictive of low mortality risk ( < 5%).^34^ Note that these patients are generally indicated for CR^30^ but would be quite uncomplicated and with a first revascularization only.

 PHC started to admit patients under the Z Benefit Package in 2013. It comprises a standardized paper-based checklist, including automatic referral and enrollment to phase 1 CR, which is activated on the third day after surgery. A CR nurse then educates the patient about CR, initiates phase I, and encourages enrollment in phase 2 (i.e., “liaison discussion”). A brochure on the CR program is given to the patient for additional information. The brochure also contains the contact details of the CR Section.

###  Design, procedure, participants and measures

 This is a retrospective cohort study involving male and female patients, 19 years and older, who underwent CABG, and were referred (i.e., completed referral form received at the CR center) to phase 2 CR at PHC from February 2013 to December 2019. There were no exclusion criteria.

 A list of post-CABG patients during the period of study was obtained from the Surgery Department. This was cross-referenced with the list of patients who enrolled in phase 2 CR obtained from the CR Section. Individual medical records were reviewed to evaluate whether the patient was referred via Z Benefit Package or not (dependent variable). The independent variable of enrollment was defined as attendance at ≥ 1 CR session. The PHC phase 2 CR program offers patients 12 sessions, delivered three times a week over one month. The attendance sheet of each patient was checked to ascertain enrollment.

 Sociodemographic and clinical characteristics (age, sex, marital status, employment status, and comorbidities) were gathered from the medical records and the electronic medical system (Medtrak).

###  Statistical analysis

 The sociodemographic and clinical characteristics of the patients were scrutinized descriptively. Frequency and proportion were used for categorical variables, and mean and standard deviation for continuous variables. Univariate and multivariate logistic regression was performed to evaluate the association between enrollment and referral strategy. An odds ratio was computed to quantify the association. Age was included as a confounder. Characteristics of patients who enrolled following automatic referral were compared to those who enrolled following referral at the discretion of the cardiologist, cardiovascular surgeon, general practitioner, or other healthcare provider through a referral letter. Student’s t-test was used to compare the two groups for continuous variables, while chi-square/Fisher exact tests were used for categorical data. The level of significance was set at 5%. Medcalc Statistical software was used to carry out the analyses.

## Results

 There were 7188 CABG patients during the period of study, of which 4792 (66.7%) were referred to CR, with 1892 (39.5%) of them referred automatically through Z Benefit Package (Table 1). Of these, 394 (8.2%) patients enrolled in phase 2 CR, with 225 (57.1%) of them referred via the Z Benefit Package automatic referral.

**Table 1:** Association of method used for referral with enrollment in phase 2 cardiac rehabilitation from 2013-2019

	**Total (N=4792)**	**Enrolled in Phase 2 (n=394; 8.2%)**	**Did Not Enroll (n=4398; 91.8%)**	**Crude OR**	**P** ^a^	**AOR**	**P** ^b^
				**(95% CI)**		**(95% CI)**	
Automatic	1892 (39.5)	225 (11.9)	1667 (37.9)	2.18 (1.8 to 2.7)	0.0001	2.19 (1.8 to 2.7)	0.0001
Usual	2900 (60.5)	169 (5.8)	2731 (62.1)				

Abbreviations: Crude OR,crude odds ratio; AOR, adjusted odds ratio (by Age); CI, confidence interval. *P* value was calculated using univariate^a^ and multivariate^b^ logistic regression

**Figure 1:**
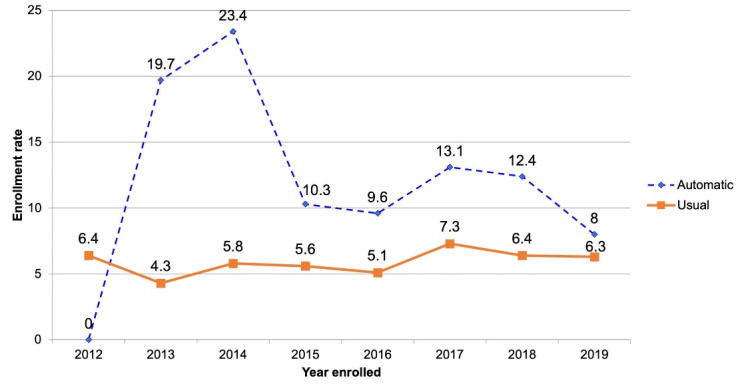
Trends in phase 2 cardiac rehabilitation enrollment by year and referral strategy Note: 2012 is baseline year. Z Benefit Package adopted at the end of 2021.

 Enrollment rate is shown by year and referral strategy in Figure 1 (see also Supplementary table S1), with 2012 data shown as a baseline. Enrollment increased significantly with introduction of the automatic referral strategy, with a peak of 23.4% in the second year after initiation. Enrollment was consistent in the usual referral group, with an average of 5.9%. There was noted a sharp decline in CR enrollment after 2014 in the automatic referral group but still consistently higher compared to the usual group. Overall, across the period of study, those who were referred via automatic referral strategy were twice more likely to enroll in phase 2 CR compared to usual care (Table 1). Even if adjusted for age, automatic referral was still significantly associated with enrolling in phase 2 CR.

Table 2 shows the characteristics of enrollees. Automatically-referred enrollees were significantly younger compared to those enrolled via the usual referral strategy. A significant difference was also observed by employment status; the majority of patients in the automatic referral group were employed. No other sociodemographic or clinical differences were observed.

**Table 2:** Sociodemographic and clinical characteristics of CR enrollees by referral strategy

**Characteristics**	**Total ** **(N=394)**	**Automatic** **Referral** **(n=225)**	**Usual referral ** **(n=169)**	**p**
**Age (mean±SD)**	59.2 ± 9.8	57.7 ± 14.0	62.4 ± 13.8	0.0010^a^
**Sex, n (%)**				
Male	331 (84.0)	190 (84.4)	141 (83.4)	0.7684^b^
Female	63 (16.0)	35 (15.6)	28 (16.6)	
**Marital Status, n (%)**				
Single	37 (9.4)	23 (10.2)	14 (8.3)	0.3273^b^
Married	326 (82.7)	188 (83.6)	138 (81.7)	
Widowed	28 (7.1)	11 (4.9)	17 (10.1)	
Separated	3 (0.8)	3 (1.3)	0 (0.0)	
**Employment Status, n (%)**				
Employed	221 (56.1)	142 (63.1)	79 (46.7)	0.0004^b^
Unemployed	26 (6.6)	16 (7.1)	10 (5.9)	
Retired	147 (37.3)	67 (29.8)	80 (47.3)	
**Comorbidities, n (%)**				
Hypertension	351 (89.1)	205 (91.1)	146 (86.4)	0.1374^b^
Diabetes	216 (54.8)	115 (51.1)	101 (59.8)	0.0881^b^
Heart Failure	66 (16.8)	34 (15.1)	32 (18.9)	0.3151^b^
PAD	9 (2.3)	5 (2.2)	4 (2.4)	> 0.9999^c^
Renal Insufficiency	6 (1.5)	2 (0.9)	4 (2.4)	0.4089^c^
COPD	15 (3.8)	7 (3.1)	8 (4.7)	0.4350^c^

Abbreviations: SD, standard deviation; PAD, peripheral arterial disease; COPD, chronic obstructive pulmonary disease. *P* values were calculated using t-test^a^ for continuous variables as well as chi-square^b^and Fisher exact test^c^ for categorical data.

## Discussion

 Many studies, including randomized trials,^35^ have demonstrated that automatic referral with CR encouragement results in significantly greater CR utilization.^7^ However, most of these studies have been performed in high-income settings, such as Canada,^11,28,29,36^ the US,^37,38^ and Australia.^39^ To our knowledge, there has only been one study on automatic referral in a non-high-income country, and it was undertaken in the Eastern Mediterranean Region.^32^ Thus, this study for the first time demonstrates the positive impact of proven CR utilization interventions in another non-high-income country, the Philippines, in Southeast Asia. Nevertheless, CR enrollment was very low overall at less than 10% of referred patients (11.9% in those automatically referred), so we must continue to mitigate CR barriers (see https://globalcardiacrehab.com/For-Patients for personalized strategies for patients).

 There are several policy implications of this work. First, it is likely interventions to promote CR utilization have not been widely implemented and evaluated in low-resource settings because there is grossly insufficient capacity to serve patients. CR programs are particularly unavailable in low and middle-income countries; only 40% of low- and middle-income countries have any programs, and there is only one spot for every 66 incident ischemic heart disease patient per year.^18-20^ The Philippines specifically has only 10 CR programs, with over 200,000 more spots needed each year to treat incident ischemic heart disease patients alone.^18^ So, it would not be ethical to institute these referral and enrollment interventions where there is no capacity. Moreover, patients often have to pay out-of-pocket for CR services in these settings;^20^ hence, automatic referral would not be workable as most patients would not be able to afford to go. Even though the criteria to qualify for the Z Benefit Package in the Philippines are somewhat narrow when compared to the patients who are indicated to attend based on evidence of benefit (e.g., no other cardiovascular procedures or previous revascularization), it is hoped more such schemes to cover CR services could be implemented in low-resource settings.

 We caution readers in over-interpreting the enrollment differences based on age, which was a benefit package-eligibility criteria. There were differences in employment status, which can impact whether patients can attend sessions which are generally offered during business hours. There is evidence that employed patients are more likely to participate in CR with plans to return to work quickly and enroll earlier into CR than non employed patients.^40^ With automatic referral, patients were referred regardless of whether they were retired or working, and more working patients thus enrolled too. It could be that when working patients are referred, they make arrangements with their employers to prioritize their recovery and enroll or they may have disability benefits after surgery. Moreover, there was a trend for greater enrollment in diabetes patients following usual referral, suggesting physicians preferentially refer them and these patients are highly motivated to attend given their greater risk. A study that compared CR outcomes in elderly cardiac patients with or without diabetes showed similar functional capacity improvements but noted higher 12 month cardiac mortality in patients with diabetes.^41^

 At this center, we shall consider ways to augment referral and encouragement for CABG patients who do not meet eligibility criteria for the Z Benefit Package yet would benefit from CR. We will also more closely examine the reasons for decay in enrollment after the second year of initiation of the CR utilization promotion intervention. It may be we need to, as other centers have done,^11,32^ leverage the electronic medical record to prompt referral, track encouragement discussions, as well as patient enrollment to promote sustainability. We should also engage the CR nurses encouraging patients about CR to understand whether they are being trained with regard to how best to go about this, and the importance. There is an evidence-based, free, open-access and brief online course certified for continuing education credits for inpatient cardiac care providers to support them in this, which also provides key discussion points (available in 5 languages here: https://globalcardiacrehab.com/CR-Utilization).^42,43^ It is also important to understand whether the nurses are given sufficient time to have these discussions with patients, and also how patients receive the discussions, so that we could optimize them and hence hopefully, patient enrollment.

 Chiefly, generalizability is limited for several reasons so caution is warranted when interpreting these results. First, with regard to design, this study is limited by its retrospective nature. With regard to measurement, the method used was chart review, hence, some data might be incomplete because of documentation issues. Moreover, we did have somewhat limited information on the characteristics of enrolling patients and we did not have detailed sociodemographic and clinical characteristics on the patients who were not referred. Second, we do not know how patients who receive CABG at PHC differ from other patients in the Philippines requiring CABG. Third, the study was undertaken at a single, tertiary care center, which has a CR program; it is likely that the impact of the utilization intervention would have been different in patients receiving CABG at other centers without a within-institution CR program.

 There are several important directions for future research stemming from this work. First, at this center, we need to understand whether patients are being equitably referred, and reasons why they are not being referred. These can be patient-related (e.g., lack of coverage, legitimate clinical contraindications, distance), but also healthcare provider-related. A study at the PHC identified financial considerations, accessibility, perceived benefit and health-care system–related aspects as factors affecting physician referral practices.^22^ Second, we need to understand why only 1 in 10 patients is enrolling despite referral, even when it is covered (e.g., distance). With this knowledge, appropriate strategies to overcome them could be instituted, such as home/electronic-based CR where covered.

 With regard to future research on CR utilization interventions in low-resource settings more broadly, first, the outcomes of CR adherence and completion were not tested; given there is now evidence automatic referral does result in greater completion in high-resource settings,^35,36^ and given the greater barriers in low-resource settings, this deserves investigation in these settings too. Prospective, multi-center studies in low-resource settings are also recommended comparing automatic versus usual referral to further determine feasibility and robustness of these findings.

## Conclusion

 A clinical pathway-based automatic CR referral strategy with CR discussion at the bedside was associated with two-times greater enrollment in Phase 2 CR after CABG, although further efforts to maintain this effect are required. While overall enrollment was still low, automatic referral may be helpful in increasing CR access even in low-resource, Southeast-Asian settings.

## Acknowledgements

 The authors would like to thank the Philippine Heart Center Cardiac Rehabilitation Section for their assistance and technical support.

## Funding

 This research did not receive any specific grant from funding agencies in the public, commercial, or not-for-profit sectors.

## Ethical approval

 The study was conducted in compliance with the ethical principles outlined in the Declaration of Helsinki and National Ethical Guidelines for Health and Health-related Research (2017). The study was reviewed and approved by the PHC Institutional Review Board, with a waiver of informed consent (PHC.IERB.01.20.13).

## Competing interests

 The authors have none to declare.

## Supplementry file

Supplementry file contains Table S1.
