# Supplementary material for: Trends in cardiac rehabilitation enrollment post-coronary artery bypass grafting upon implementation of automatic referral in Southeast Asia: A retrospective cohort study
Source: J Cardiovasc Thorac Res. 2022 Jun 28;14(2):84–9. doi: 10.34172/jcvtr.2022.22 (PMC9339729; doi:10.34172/jcvtr.2022.22)
Supplement: Supplementary file 1 — Supplementry file contains Table S1. [file jcvtr-14-84-s001.pdf]

Supplementary Table S1: Post-CABG Enrollment by Referral Strategy and Year

Year	Number of CABG Procedures (n=7188)	Number of Referred Patients		CR Utilization			
				Z-Ben		Usual	
		Z-Ben	Usual	Enrolled	Not Enrolled	Enrolled	Not Enrolled
2012*	793	0	501	0	0	32	469
2013	871	61	445	12	49	19	426
2014	883	141	396	33	108	23	373
2015	932	234	373	24	210	21	352
2016	1090	322	412	31	291	21	391
2017	1111	352	424	46	306	31	393
2018	1206	380	450	47	333	29	421
2019	1095	402	400	32	370	25	375

*Baseline Year
